# The application of THz-TDS in the characterization of Bayan Obo magnetite ore composition

**DOI:** 10.1038/s41598-024-65772-0

**Published:** 2024-07-01

**Authors:** Siqi Zhang, Zhiyuan Zheng, Mingrui Zhang, Tong Zhang, Zili Zhang, Haochong Huang

**Affiliations:** School of Science, China University of Geosciences (Beijing), Beijing, 100083 China

**Keywords:** Bayan Obo, Magnetite, THz-TDS, Absorption coefficient, Random forest, Geophysics, Applied optics, Infrared spectroscopy

## Abstract

The application of terahertz time-domain spectroscopy (THz-TDS) in the quantitative analysis of major minerals in Bayan Obo magnetite ore was explored. The positive correlation between the optical parameters of the original ore and its iron content is confirmed. The detections of three main iron containing minerals, including magnetite, pyrite, and hematite, were simulated using corresponding reagents. The random forest algorithm is used for quantitative analysis, and FeS_2_ is detected with precision of R^2^ = 0.7686 and MAE = 0.6307% in ternary mixtures. The experimental results demonstrate that THz-TDS can distinguish specific iron containing minerals and reveal the potential application value of this testing method in exploration and mineral processing fields.

## Introduction

Terahertz (THz) wave is electromagnetic wave in a specific frequency band. Its frequency ranges from 0.1 to 10 THz (1 T = 1 × 10^12^), and its wavelength is at 30 μm–3 mm. THz waves are located between infrared and microwave in the electromagnetic spectrum. Since the 1990s, THz devices have developed rapidly, and THz technology, as a key technology in the future, has become a research hotspot. THz waves have unique advantages such as low energy, high penetration, fingerprint, and high bandwidth^1,2^. Currently, THz technology has been widely applied in various fields such as biology, materials, medicine, communication, and non-destructive testing^3–8^.

In the past decade, multiple research groups have conducted characterization studies on rocks and minerals using THz technology, and attempted to explain the information reflected by the mineral samples’ optical parameters in the THz band. Some researchers use computer algorithms, including machine learning algorithms, to analyze terahertz spectral data. At present, THz spectroscopy has produced some valuable results in fields such as petroleum^9^, coal^10,11^, and water containing minerals^12–16^, and some minerals with characteristic absorption peaks in the THz band have been discovered^17^. However, most common inorganic minerals have no absorption peaks in the THz band, and their absorption coefficients for THz waves are generally low^18^. There is few quantitative researches on these minerals based on THz spectra.

The Bayan Obo mining area is located in Baotou City, Inner Mongolia Autonomous Region, China. This area has the world's largest light rare earth deposit and the second largest niobium (Nb) deposit, as well as a large iron ore deposit. The ore-forming process of the Bayan Obo mining area is very complex, and it has the characteristics of high element and mineral composition, low grade, fine particle size of useful minerals, and diverse types of ores, which brings great difficulties to the exploration, recovery, and utilization of resources^19–21^.

Presently, chemical methods are used in actual production to determine the iron content in ores, which have high accuracy but only determine the element content. Some traditional physical characterization methods—for example, X-ray analysis, scanning electron microscope, Raman spectroscopy—have been used to analyze the composition of ores, but they also have disadvantages such as low efficiency, high cost, and insufficient accuracy^22,23^. THz time-domain spectroscopy (THz-TDS), as an efficient non-destructive testing method, has been proven to be able to distinguish some common iron containing substances^24^. Therefore, THz-TDS also has the potential to be applied in iron ore exploration.

This study used THz-TDS to analyze core powder samples and reagents corresponding to some major minerals in the Bayan Obo magnetite ore. The random forest (RF) algorithm was used to process spectral data. The optical response of different mineral components in the THz band is summarized, and the application value of THz technology in physical exploration and mineral processing is preliminarily explored.

## Results

### Optical parameters of original ore samples

The absorption coefficient and refractive index curves of all original ore samples have no absorption peaks or obvious extreme points in the 0.2–1.3 THz band. The optical parameters of original ore samples are mainly affected by TFe.

**Figure 1:**
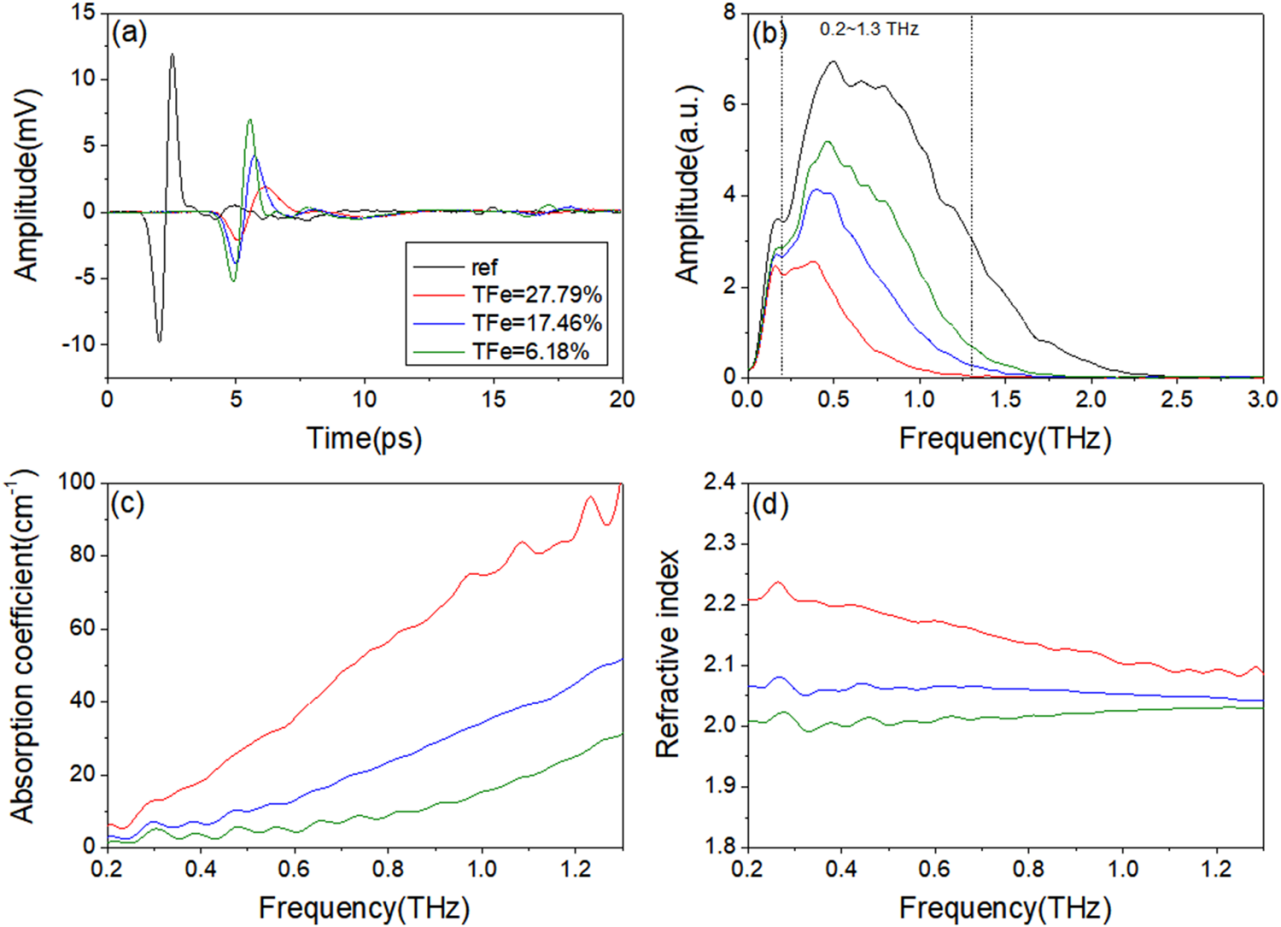
THz-TDS data graphs of three original ore samples with different iron contents. (**a**) Time domain spectrum. (**b**) Frequency spectrum. (**c**) Absorption coefficient. (**d**) Refractive index.

Figure 1 shows the THz-TDS test results of three samples with TFe of 27.79%, 17.46%, and 6.18%. From the time domain spectrum curves, all three samples have a significant attenuation and delay effect on the pulse THz wave, with higher TFe being more pronounced. The frequency spectrum and absorption coefficient curves indicate that the absorption coefficients of all samples increase with increasing frequency, and the ratio of absorption coefficients of the three samples at the same frequency is approximately 4:2:1 from high to low according to TFe. The difference in refractive index values is relatively small. Samples with higher TFe have higher refractive index values, which tend to decrease with increasing frequency. Samples with lower TFe have generally stable refractive index values in the THz frequency band.

**Figure 2:**
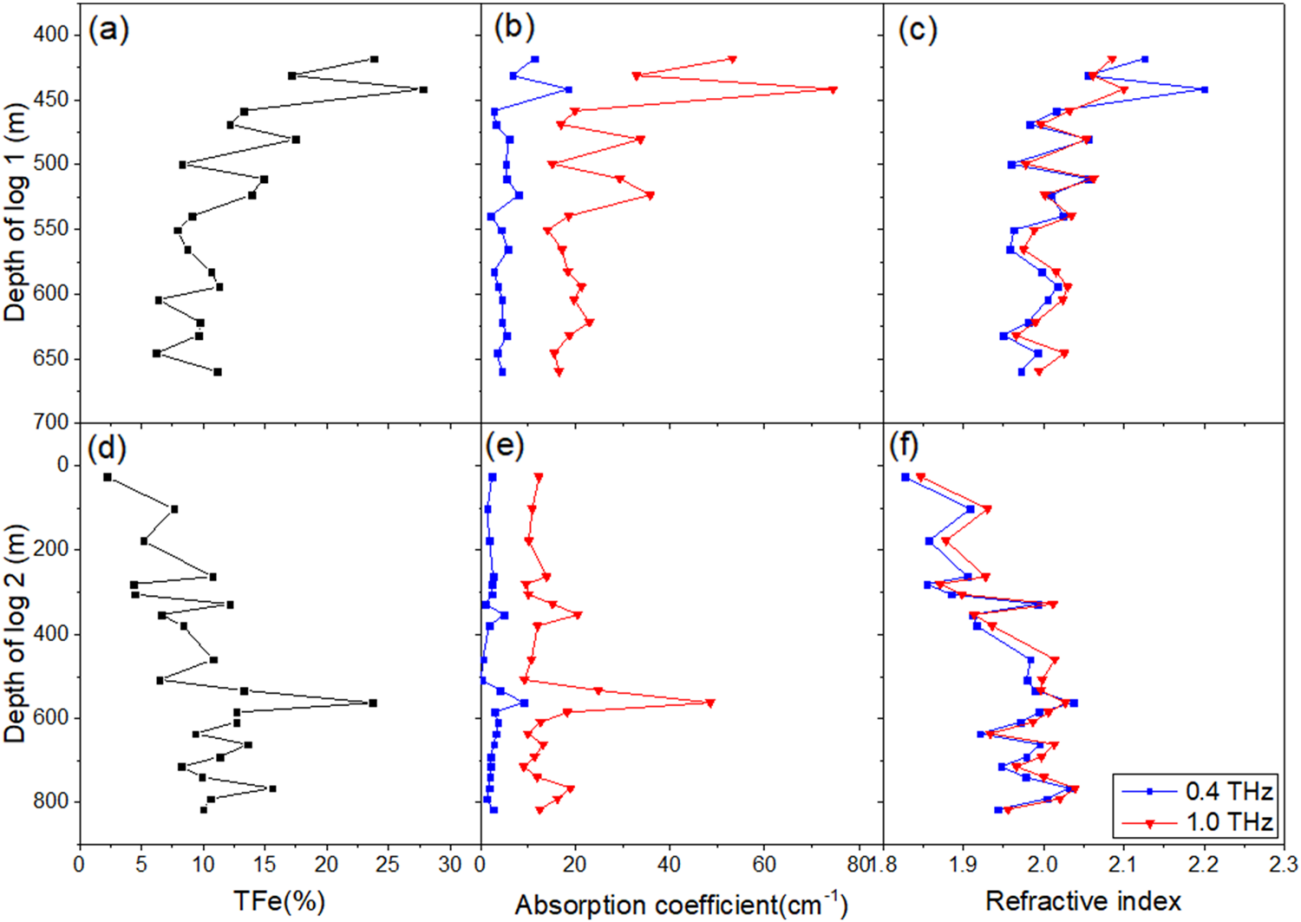
Line chart of TFe and optical parameters at specific frequencies of original ore samples. (**a**) Log 1 TFe. (**b**) Log 1 absorption coefficient. (**c**) Log 1 refractive index. (**d**) Log 2 TFe. (**e**) Log 2 absorption coefficient. (**f**) Log 2 refractive index.

**Figure 3:**
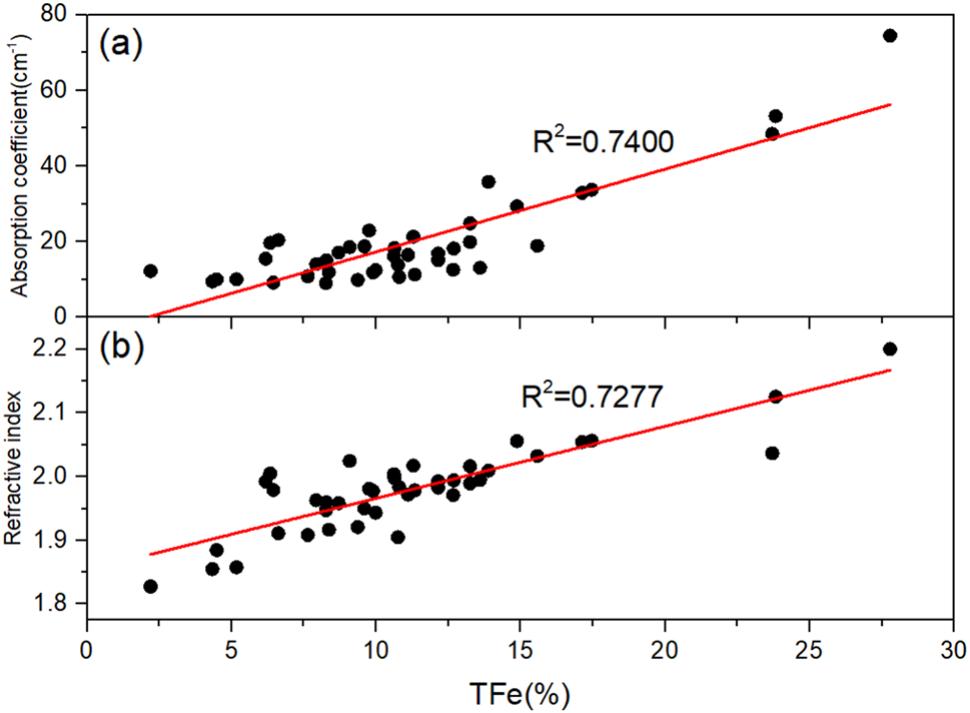
Scatter plots of absorption coefficient and refractive index of original ore samples. (**a**) 1.0 THz absorption coefficient. (**b**) 0.4 THz refractive index.

Due to the monotonic trend of the original ore curve, the absorption coefficient and refractive index values at frequencies of 0.4 THz and 1.0 THz are taken as line graphs to visually reflect the correspondence between optical parameters and TFe values, as shown in Fig. 2. It can be seen that the two optical parameters of the original ore correspond well to the iron content. The 1.0 THz absorption coefficient and 0.4 THz refractive index data with significant numerical fluctuations are used as scatter plots for a linear fit, as shown in Fig. 3. The R^2^ values of the fitted lines are all above 0.7, and the R^2^ value of the absorption coefficient are slightly higher than that of the refractive index. The relationship shown in the figures between the optical parameters in the THz band and the iron content of Bayan Obo magnetite is consistent with the research of Zhang et al.^25^

### Optical parameters of reagent samples

The results above confirm the resolution ability of THz-TDS to the iron content of the original ore. The optical parameters in the THz frequency band can be used to detect iron containing minerals in the original ore samples of the Bayan Obo. Three main iron containing minerals—magnetite, pyrite and hematite—were selected from the original ore, simulated samples were made by corresponding reagents—Fe_3_O_4_, FeS_2_ and Fe_2_O_3_, to explore the resolution ability of THz-TDS for a single mineral.

Among the selected minerals, magnetite is the main resource mineral in the origin ore. Pyrite is the main harmful impurity, as its sulfur element can damage equipment and products. Hematite is a common usable impurity, but its optical parameters may affect the accuracy of optical methods in determining the content of pyrite.

**Figure 4:**
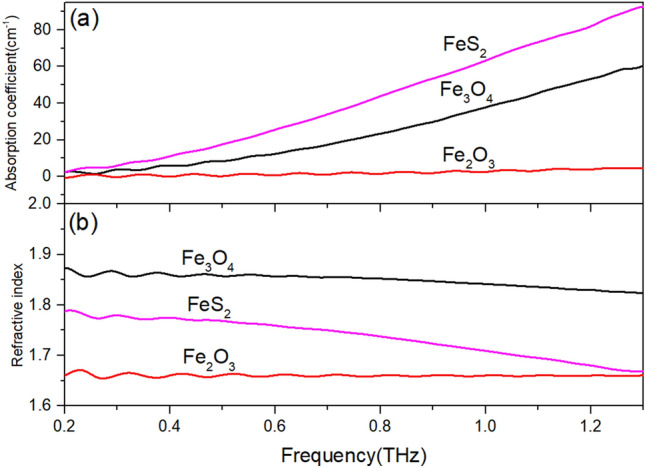
(**a**) Absorption coefficient and (**b**) refractive index of the 20% mass fraction reagent samples.

Figure 4 shows the optical parameters of the three reagents mentioned above in the THz band. The absorption coefficients of the three reagent samples are significantly different. Taking the values at 1.0 THz as a typical frequency, the absorption coefficients of FeS_2_ sample is 62.7190 cm^−1^, the Fe_3_O_4_ sample is 37.3639 cm^−1^, and the Fe_2_O_3_ sample is 2.0600 cm^−1^. The absorption coefficient of Fe_2_O_3_ sample is one order of magnitude lower than that of the other two samples.

The refractive index values of the three reagents are also different in the low frequency band. Taking the value at 0.4 THz a typical frequency, the refractive index of Fe_3_O_4_ sample is 1.8552, FeS_2_ sample is 1.7751, and Fe_2_O_3_ sample is 1.6645. However, in the high frequency band, the refractive index of FeS_2_ sample decreases to a level similar to that of Fe_2_O_3_ sample, while the other two reagent samples show smaller changes.

As iron oxides, there is a significant difference in the optical parameters between Fe_3_O_4_ and Fe_2_O_3_, which proves that mineral content rather than element content determines the optical parameters of rock samples in the THz band. In the following experiments, the three reagents were mixed with each other in different proportions to prepare samples, simulating the iron concentrate after magnetic separation in actual production.

**Figure 5:**
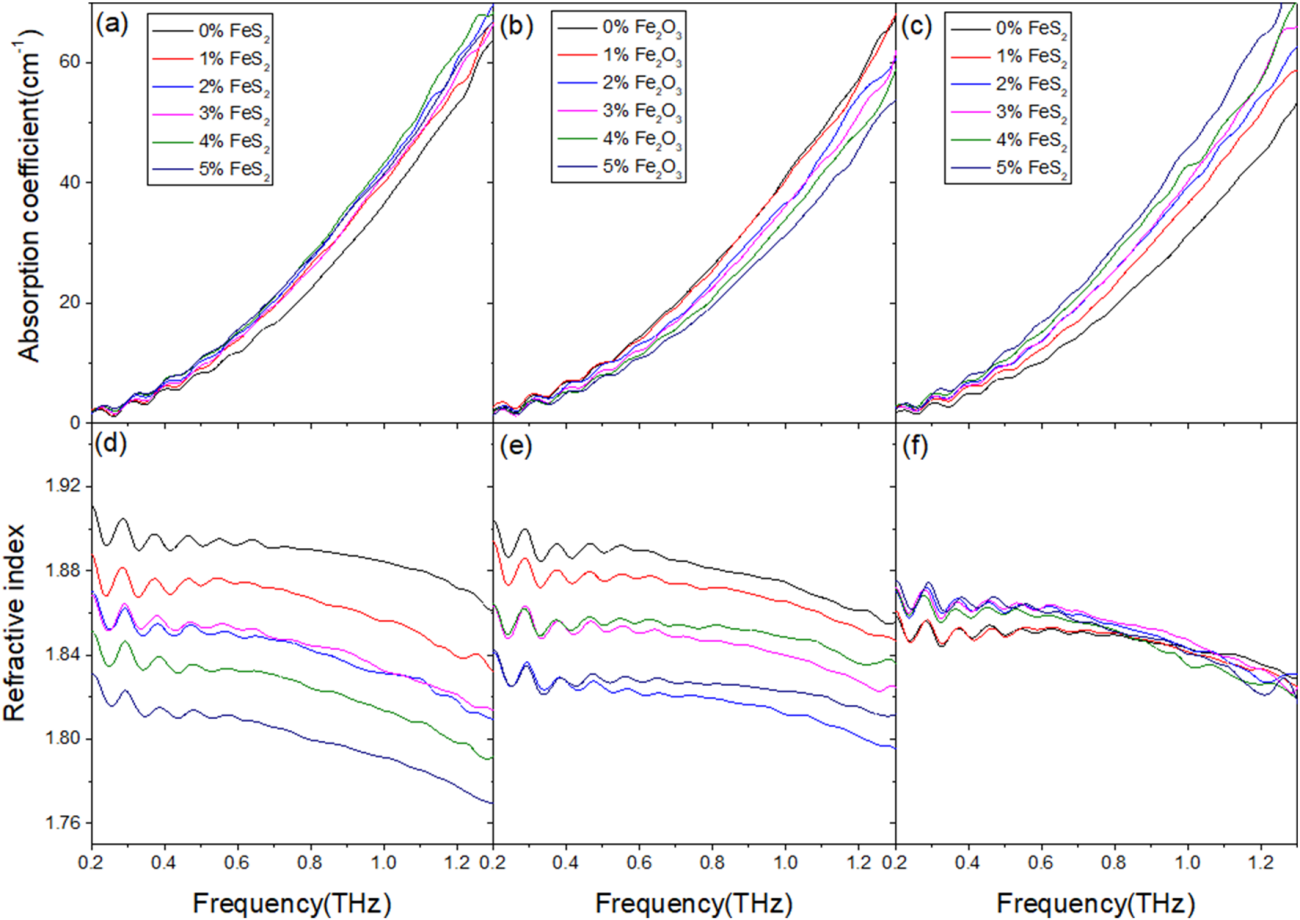
Absorption coefficient and refractive index of mixed reagent samples. (**a**) Absorption coefficient of FeS_2_-Fe_3_O_4_ samples. (**b**) Absorption coefficient of Fe_2_O_3_-Fe_3_O_4_ samples. (**c**) Absorption coefficient of FeS_2_-Fe_2_O_3_-Fe_3_O_4_ samples. (**d**) Refractive index of FeS_2_-Fe_3_O_4_ samples. (**e**) Refractive index of Fe_2_O_3_-Fe_3_O_4_ samples. (**f**) Refractive index of FeS_2_-Fe_2_O_3_-Fe_3_O_4_ samples.

The test results of mixed reagent samples with different proportions are shown in Fig. 5. Due to the large number of samples and measurements, the curves only contains partial data. The images of three sets of samples reflect the response of THz-TDS to proportion changes of FeS_2_-Fe_3_O_4_, Fe_2_O_3_-Fe_3_O_4_, and Fe_2_O_3_-FeS_2_ in iron containing samples dominated by Fe_3_O_4_. According to the effective medium theory, the optical parameters of mixed samples are determined by the proportion of different components and their respective optical parameters. The general trend of the changes in each group of curves is consistent with this conclusion, but there is a certain degree of fluctuation in the specific values, especially in refractive index curves where the numerical differences are not significant. This indicates that in practice, the accuracy of predicting sample composition through optical parameters is affected by various random errors.

### Random forest algorithm for predicting sample composition

In order to utilize all the information of optical parameter curves and establish the correspondence between the material composition and optical parameters more accurately, it is necessary to introduce computer algorithms for assistance.

Absorption coefficient and refractive index data of three series of mixed reagent samples were used as input data to the random forest algorithm to predict the substance content values. The training and prediction sets were determined by the random seed, with 80% of the data being the training set and 20% being the prediction set. Table 1 shows the prediction errors. From the prediction results based on absorption coefficient data, the coefficients of determination (R^2^) for each type of samples are above 0.9, with the mean squared error (MSE) and the mean absolute error (MAE) values below 0.5. A higher R^2^ value indicates a good correlation between absorption coefficient data and substance content values, while lower MSE and MAE values indicate lower prediction errors. The prediction results based on refractive index data are much worse, with lower R^2^, higher MSE and MAE. This is because the relative difference in refractive index values between the three iron containing substances is smaller than that in absorption coefficient values.

**Table 1:** Prediction error table of random forest algorithm.

Sample type	Optical parameters	R^2^	MSE (%^2^)	MAE (%)
FeS_2_-Fe_3_O_4_	Absorption coefficient	0.9478	0.2163	0.3477
	Refractive index	0.3310	1.7472	1.1764
Fe_2_O_3_-Fe_3_O_4_	Absorption coefficient	0.9509	0.3928	0.4636
	Refractive index	0.5957	1.0559	0.8455
FeS_2_-Fe_2_O_3_-Fe_3_O_4_	Absorption coefficient	0.9494	0.1439	0.2682
	Refractive index	0.6898	1.1563	0.9318

**Figure 6:**
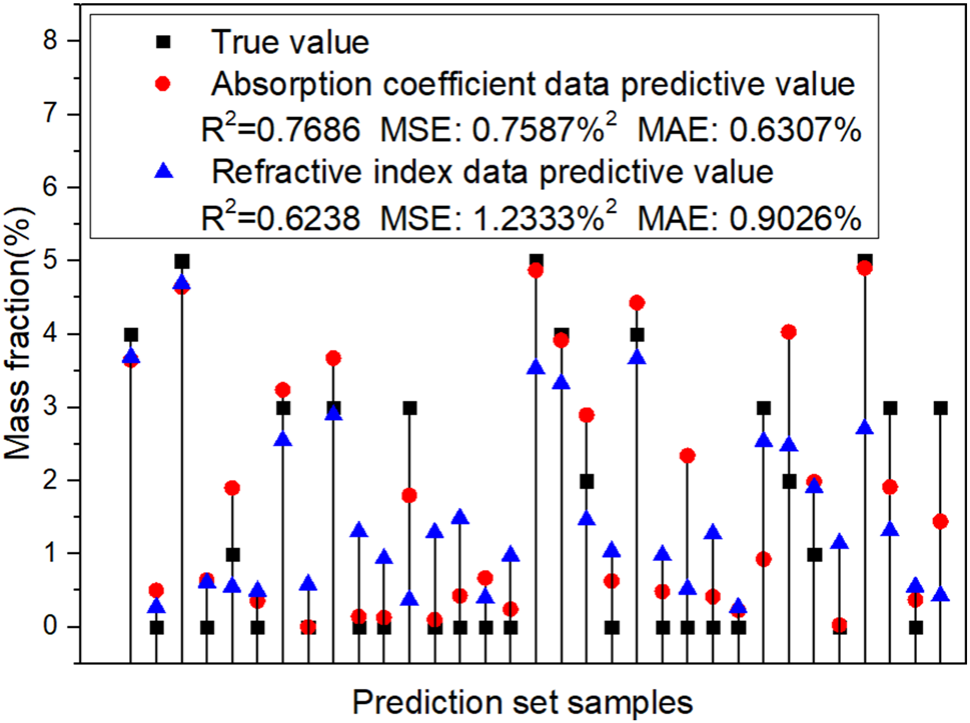
FeS_2_ content prediction results of all mixed reagent samples. The data used in the algorithm are (**a**) absorption coefficient and (**b**) refractive index.

The prediction results indicate that the THz spectral data has a precise response to the changes in the composition of binary iron containing substances. Further testing will be conducted on ternary substances. Predict the mass fraction of FeS_2_ using data from all 162 sets of mixed reagent samples. The prediction results are shown in Fig. 6.

According to the absorption coefficient data prediction results of ternary samples, the R^2^ value is 0.7686, the MSE value is 0.7587%^2^, and the MAE value is 0.6307%. The prediction error based on refractive index data is still slightly higher. Calculated with a MAE of 0.6307% for FeS_2_, the MAE for S element is as low as 0.3364%.

The results of the two optical parameters are compared with each other, the accuracy of using absorption coefficient data for prediction is slightly higher than that of refractive index data. The predicted results of FeS_2_ and sulfur content based on all reagent samples’ absorption coefficient data are shown in Table 2. The prediction error of S element mass fraction in over 90% (30 out of 33) of samples is less than 1%. The results indicates that the THz-TDS test has great potential application value in the field of detecting sulfur content in magnetite concentrates.

**Table 2:** Content prediction error based on absorption coefficient data for each prediction set sample (Unit: %).

	1	2	3	4	5	6	7	8	9	10	11	12	13	14	15	16	17
FeS_2_	0.36	0.50	0.36	0.64	0.90	0.36	0.24	0.00	0.67	0.14	0.13	1.20	0.10	0.43	0.67	0.24	0.13
S	0.19	0.27	0.19	0.34	0.48	0.19	0.13	0.00	0.36	0.08	0.07	0.64	0.05	0.23	0.36	0.13	0.07

	18	19	20	21	22	23	24	25	26	27	28	29	30	31	32	33	
FeS_2_	0.09	0.90	0.63	0.43	0.49	2.34	0.41	0.23	2.07	2.03	0.99	0.03	0.10	1.09	0.37	1.56	
S	0.05	0.48	0.34	0.23	0.26	1.25	0.22	0.12	1.10	1.08	0.53	0.02	0.05	0.58	0.20	0.83	

## Discussion

This study obtains a series of experimental data through the THz-TDS method and comes to the following conclusions:

- The THz-TDS method has good discrimination ability between the main iron containing minerals and non-iron containing minerals in the Bayan Obo REE-Nb-Fe ore, and the optical parameters in the THz frequency band are significantly correlated with the iron content of the original ore.
- The THz-TDS method has certain discriminative ability for mixing iron containing reagents—Fe_3_O_4_, Fe_2_O_3_ and FeS_2_—in different proportions. This proves that this method can detect mineral content rather than simply elemental content in rock samples.
- The mixing ratios of the samples of binary and ternary components were predicted using the random forest algorithm. The R^2^ value for predicting FeS_2_ content in ternary samples is 0.7686, and the MAE value is 0.6307%. The result is expected to be used for the detection of real minerals.

The research results indicate that the THz-TDS method has varying degrees of resolution ability for various major iron containing minerals in Bayan Obo. In order for this new method to be effective in practical production, further research is needed in the following areas:

- The detection results obtained by the THz-TDS method should be compared and combined with established mineralogical methods to consolidate the effectiveness of the test results and improve detection accuracy.
- More experiments are needed on other metallic and non-metallic minerals to establish a comprehensive mineral spectral database, in order to expand the application scope of THz-TDS in the mineral field.
- Based on validated composite detection methods and a mineral spectral database, the detection of samples with more complex compositions and closer proximity to real rocks should be achieved.
- Other necessary research, including program standardization, algorithm optimization, simplification of sample preparation process, etc.

In summary, the THz-TDS method is expected to serve as a cost-effective and efficient new characterization method to make magnetite exploration and beneficiation processes faster and more convenient after thorough research and rigorous validation.

## Methods

### Experimental samples

The original ore samples were taken from the Bayan Obo rare iron-earth-niobium (Fe-REE-Nb) ore body. The main rock types are dolomite, rare earth dolomite, dolomite type magnetite ore, carbonaceous slate, siliceous slate, etc. The main mineral components include fluorite, dolomite, aegirine, magnetite, pyrite, hematite, etc. Samples were taken from two log wells (referred to as log 1 and log 2) located far apart. The rock cores taken from the two boreholes were divided into sections of 2–3 m, and they were crushed and mixed to represent the average mineral composition of the core section. Approximately 20 sets of samples were taken at similar intervals from the core powder samples of each borehole for THz analysis. The mass fraction of iron (TFe) of each sample was measured by chemical methods. The 200 mesh powder of the original ore samples were mixed with polytetrafluoroethylene (PTFE) powder in a mass ratio of 1:1 to prepare the test sample. Took 0.3 g of powder from each sample and pressed for 2 min under a pressure of 4 tons to produce a circular sample with a diameter of 13 mm.

Three samples with TFe of 27.79%, 17.46%, and 6.18% were selected from all the original ore samples as representatives. TFe and optical parameter curves of all original ore samples can be found as Supplementary Table S1 and Supplementary Figure S1 online. Table 3 shows the X-ray fluorescence spectroscopy (XRF) test results of the three samples. The metal elements with high content in the samples include Fe, Ca, Mg and Mn. There are also other metal elements such as Ce, Na, Ba, etc. Since XRF does not detect elements with atomic numbers lower than F, the test data is only the relative proportion of each element rather than their actual mass fraction in the original ore.

**Table 3:** XRF detection results on three representative samples.

	Fe %	Ca %	Mg %	Mn %	Si %	Ce %	Na %	Ba %
Sample 1	41.069	40.719	6.099	5.370	1.717	1.140	0.303	0.316
Sample 2	26.672	51.583	6.960	5.355	0.722	1.843	0.185	0.149
Sample 3	16.649	51.151	6.127	4.166	9.652	1.338	1.567	1.345

In order to test the characterization ability of THz-TDS for individual iron containing minerals, 200 mesh reagent powders of three different substances, including Fe_3_O_4_ (corresponding to magnetite), FeS_2_ (corresponding to pyrite), and Fe_2_O_3_ (corresponding to hematite), were selected for sample preparation. Reagent samples were divided into four series:

- Each reagent was mixed with PTFE in a mass ratio of 25:75 to simulate the optical parameters of pure minerals in the THz band.
- Mixed FeS_2_ reagent and Fe_3_O_4_ reagent in different proportions, and then mixed the mixture with PTFE in a mass ratio of 20:80. The proportion of FeS_2_ was 0%, 1%, 2%, 3%, 4%, 5%.
- Mixed Fe_2_O_3_ reagent and Fe_3_O_4_ reagent in different proportions, and then mixed the mixture with PTFE in a mass ratio of 20:80. The proportion of Fe_2_O_3_ was 0%, 1%, 2%, 3%, 4%, 5%.
- Mixed FeS_2_ reagent and Fe_2_O_3_ reagent in different proportions, and then mixed the mixture with Fe_3_O_4_ and PTFE in a mass ratio of 5:15:80. The proportion of FeS_2_ was 0%, 1%, 2%, 3%, 4%, 5%.

The pressing method of the reagent samples was the same as that of the original ore samples mentioned above. By preparing multiple samples and conducting multiple measurements, ensure that each proportion has 9 sets of measurement data for algorithm training.

### Terahertz time-domain spectroscopy

**Figure 7:**
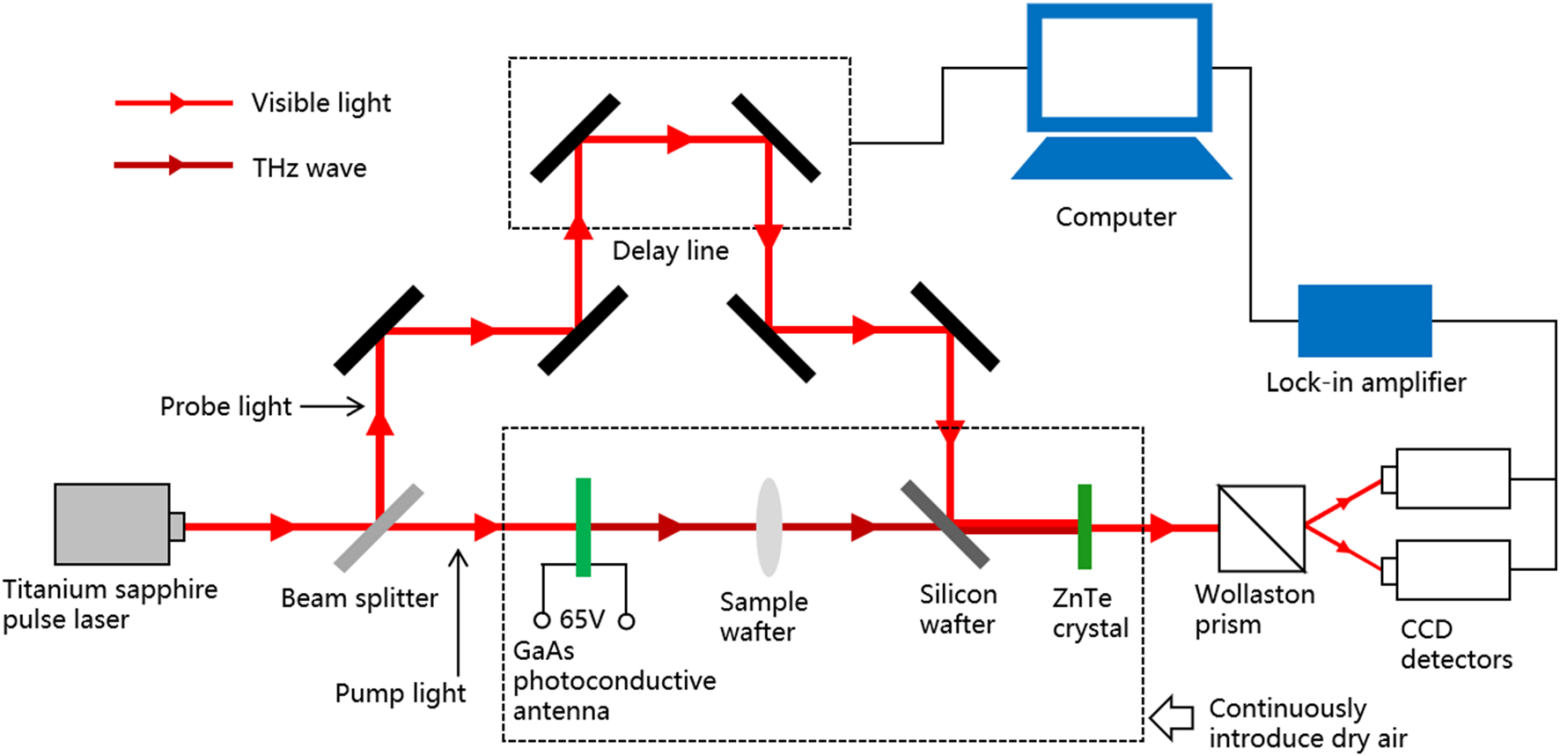
Schematic diagram of THz-TDS used in the experiment.

The main data in the experiment was obtained by a THz time-domain spectrometer shown in Fig. 7. The pulsed laser generated by a titanium sapphire laser is divided into pump light and probe light. The pump light is irradiated onto the GaAs photoconductive antenna and converted into THz waves. Passing THz waves through the sample wafer can obtain the optical parameters of the sample in the THz band. The pump light is converted into visible light through ZnTe crystals, collinear with the probe light and then enters the charge coupled devices (CCD) detectors. By adjusting the delay of the probe light, time scanning can be performed on the THz signal passing through the sample. Based on the frequency distribution of the incident THz signal, the optical properties of the sample in the 0.2–1.3 THz band can be collected with high quality.

### Extraction of optical parameters

The original signal is the time-domain spectrum. Frequency spectrum can be obtained through fast Fourier transform (FFT). Absorption coefficient and refractive index of the signal are obtained through algorithms. These algorithms conforms to the physical model proposed by T.D. Dorney and L. Duvillaret et al. for the optical parameters of materials in the THz band^26,27^. Assuming the sample is a thin plate with two parallel sides, and THz waves are vertically incident, the extinction coefficient of the sample *κ* is far less than the refractive index *n*, the refractive index of air is 1, and the refractive index *n(ω)* and absorption coefficient *α(ω)* can be obtained by Eqs. (1) and (2).

1 $$n(\omega ) = \varphi (\omega )\frac{c}{\omega d} + 1$$

2 $$\alpha (\omega ) = \frac{2}{d}\ln \left\{ {\frac{4n(\omega )}{{\rho (\omega )[n(\omega ) + 1]^{2} }}} \right\}$$

In the two equations, $\Phi (\omega )$ is the phase difference between the sample signal and the reference signal, *d* is the thickness of the sample, and *c* is the velocity of light in vacuum, $\rho (\omega )$ is the ratio of modulus of sample signal and reference signal.

### Random forest algorithm

To further analyze spectral information and conduct precise qualitative research on the relationship between optical parameters and substance content, the random forest algorithm was used to process the experimental data of reagent samples.

In the case of unknown magnetic permeability values, the dielectric constant cannot be calculated from the complex refractive index. Therefore, the random forest algorithm was used to qualitatively analyze the relationship between optical parameters and material content without any physical models.

Random forest is an efficient machine learning algorithm that can be applied to classification problems, regression problems, and feature selection problems^28,29^. Random forest is a predictor composed of a random basic regression tree in the form of *{r*_*n*_*(X, θ*_*m*_*, D*_*n*_*), m* ≥ *1}*, where *θ*_*m*_ is the independent and identically distributed output of the random variable *θ*, *D*_*n*_ is the given dataset. These basic regression trees are combined to form aggregated regression estimates. The loss function between the predicted output value and the true value under input *X* using Eq. (3).

3 $$\overline{r}_{n} \left( {X,D_{n} } \right) = E_{\theta } \left[ {r_{n} \left( {X,\theta ,D_{n} } \right)} \right]$$

In Eq. (3), *E*_*θ*_ is the expectation for random parameters. Each random tree outputs the average of all *Y*_*i*_, for which the corresponding vector *X*_*i*_ falls in the same unit as *X*. As a convention, the estimated value for empty cells is set to 0, and finally the expected value for parameter *θ* is taken*.* The form of regression estimation using the random forest algorithm is Eq. (4).

4 $$\overline{r}_{n} \left( X \right) = E_{\theta } \left[ {r_{n} \left( {X,\theta } \right)} \right] = E_{\theta } \left[ {\frac{{\sum\nolimits_{i = 1}^{n} {Y_{i} 1_{{\left[ {X_{i} \in A_{n} \left( {X,\theta } \right)} \right]}} } }}{{\sum\nolimits_{i = 1}^{n} {X_{i} 1_{{\left[ {X_{i} \in A_{n} \left( {X,\theta } \right)} \right]}} } }}1_{{E_{n} \left( {X,\theta } \right)}} } \right]$$

The algorithm randomly selects about 10% of the input sample data as the prediction set to simulate and predict the content of the target substance. The prediction results of the algorithm are evaluated through three parameters: coefficients of determination (R^2^), mean squared error (MSE) and the mean absolute error (MAE):

5 $$R^{2} = 1 - \frac{SSR}{{SST}}$$

6 $$MSE = \frac{1}{n}\sum\limits_{i = 1}^{n} {(\overline{y}_{i} - y_{i} )^{2} }$$

7 $$MAE = \frac{1}{n}\sum\limits_{i = 1}^{n} {\left| {\overline{y}_{i} - y_{i} } \right|}$$

In Eq. (5), SSR is the sum of squared residuals, SST is the total sum of squares. In Eqs. (6) and (7), *ȳ*_*i*_ is the predicted value of the i-th group of data in the prediction set, while *y*_*i*_ is the corresponding true value. R^2^ represents the correlation between substance content and optical parameters (up to 1), MSE and MAE represent the overall prediction error. A good prediction result requires a high R^2^ value while low MSE and MAE values^25^.

### Supplementary Information

Supplementary Information.

## Data Availability

The datasets generated and analysed during the current study are available from the corresponding author on reasonable request.

## Abbreviations

THz: Terahertz
THz-TDS: Terahertz time-domain spectroscopy
PTFE: Polytetrafluoroethylene
FFT: Fast Fourier transform
RF: Random forest
Fe-REE-Nb: Iron-rare earth-niobium
MSE: Mean squared error
MAE: Mean absolute error
