# Supplementary material for: The application of THz-TDS in the characterization of Bayan Obo magnetite ore composition
Source: Sci Rep. 2024 Jul 1;14:15033. doi: 10.1038/s41598-024-65772-0 (PMC11217283; doi:10.1038/s41598-024-65772-0)
Supplement: Supplementary file 1 — Supplementary Information. [file 41598_2024_65772_MOESM1_ESM.docx]

**Supplementary Information:**

（Title of the manuscript: The Application of THz-TDS in the Characterization of Bayan Obo Magnetite Ore Composition.

Authors: Siqi Zhang, Zhiyuan Zheng, Mingrui Zhang, Tong Zhang, Zili Zhang, Haochong Huang.）

To avoid redundant information, the main text does not display information on all original ore samples. Table S1 and Figure S1 are supplementary information.

**Supplementary Table S1.** TFe table of original ore samples

| **Sample**  **name** | **Average depth (m)** | **TFe (%)** | **Sample name** | **Average depth (m)** | **TFe (%)** |
| --- | --- | --- | --- | --- | --- |
| Log 1-161 | 418.00 | 23.83 | Log 2-11 | 25.60 | 2.20 |
| Log 1-166 | 431.20 | 17.13 | Log 2-41 | 101.70 | 7.64 |
| Log 1-170 | 441.55 | 27.79 | Log 2-71 | 177.50 | 5.17 |
| Log 1-176 | 458.40 | 13.25 | Log 2-103 | 262.60 | 10.76 |
| Log 1-180 | 468.90 | 12.15 | Log 2-110 | 280.05 | 4.34 |
| Log 1-184 | 480.40 | 17.46 | Log 2-120 | 305.40 | 4.49 |
| Log 1-191 | 499.40 | 8.28 | Log 2-130 | 327.40 | 12.15 |
| Log 1-195 | 510.75 | 14.88 | Log 2-140 | 353.15 | 6.62 |
| Log 1-200 | 523.30 | 13.89 | Log 2-150 | 379.65 | 8.37 |
| Log 1-206 | 539.65 | 9.09 | Log 2-182 | 460.10 | 10.80 |
| Log 1-210 | 550.50 | 7.93 | Log 2-201 | 508.10 | 6.45 |
| Log 1-216 | 565.40 | 8.71 | Log 2-211 | 532.95 | 13.25 |
| Log 1-222 | 582.40 | 10.64 | Log 2-222 | 562.40 | 23.71 |
| Log 1-226 | 594.40 | 11.29 | Log 2-231 | 585.30 | 12.69 |
| Log 1-230 | 604.30 | 6.34 | Log 2-240 | 608.40 | 12.67 |
| Log 1-236 | 621.60 | 9.76 | Log 2-251 | 635.75 | 9.37 |
| Log 1-240 | 631.90 | 9.60 | Log 2-261 | 661.20 | 13.60 |
| Log 1-245 | 645.60 | 6.18 | Log 2-272 | 691.05 | 11.34 |
| Log 1-250 | 659.60 | 11.11 | Log 2-281 | 714.20 | 8.27 |
|  |  |  | Log 2-291 | 739.30 | 9.90 |
|  |  |  | Log 2-301 | 765.60 | 15.58 |
|  |  |  | Log 2-311 | 790.30 | 10.62 |
|  |  |  | Log 2-321 | 816.15 | 9.99 |


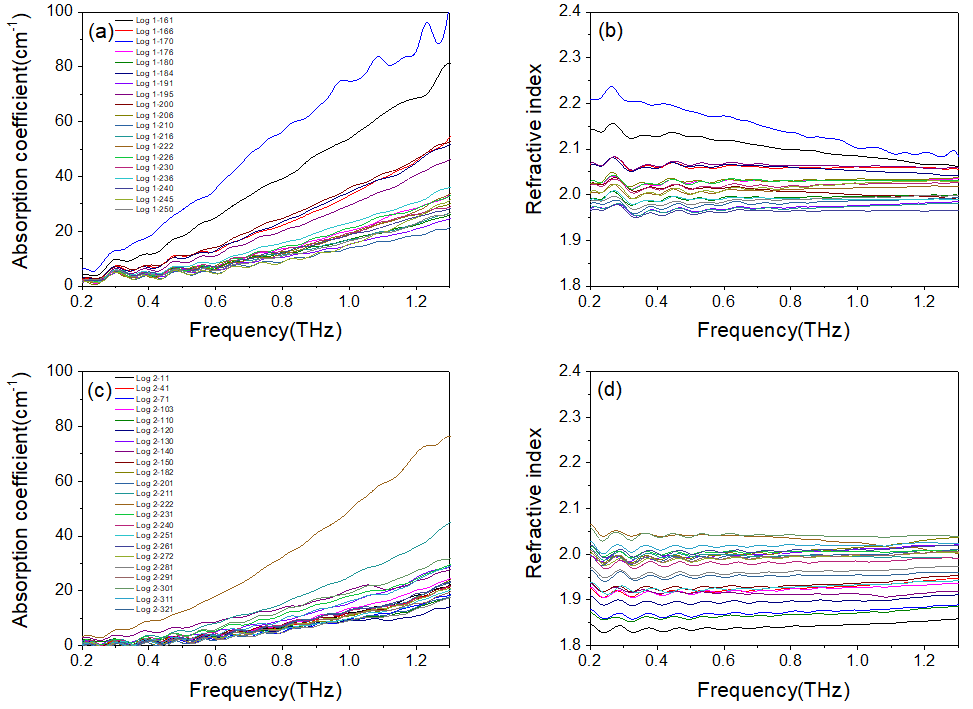


**Supplementary Figure S1.** THz-TDS data graphs all original ore samples. (a) Log 1 absorption coefficient. (b) Log 1 refractive index. (c) Log 2 absorption coefficient. (d) Log 2 refractive index.
